# Extreme gradient boosting using conventional parameters accurately predicts insulin sensitivity in young and middle-aged Japanese persons

**DOI:** 10.3389/fendo.2025.1661376

**Published:** 2025-10-17

**Authors:** Norimitsu Murai, Naoko Saito, Sayuri Nii, Hiroto Nishikawa, Eriko Kodama, Tatsuya Iida, Hideyuki Imai, Mai Hashizume, Rie Tadokoro, Chiho Sugisawa, Toru Iizaka, Fumiko Otsuka, Shun Ishibashi, Shoichiro Nagasaka

**Affiliations:** ^1^ Division of Diabetes, Metabolism and Endocrinology, Showa Medical University Fujigaoka Hospital, Yokohama, Japan; ^2^ Division of Endocrinology and Metabolism, Department of Medicine, Jichi Medical University, Shimotsuke, Japan

**Keywords:** oral glucose tolerance test, insulin sensitivity, triglyceride glucose index, machine learning, extreme gradient boosting

## Abstract

**Background:**

This study tested the hypothesis that insulin sensitivity (SI) can be estimated using machine learning (ML) based only on physical indicators or with the addition of lipid and fasting glucose levels.

**Methods:**

In 1,268 young (age <40 years, normal glucose tolerance; NGT) and 1,723 middle-aged Japanese persons with NGT (n=1,276) and glucose intolerance (n=447), the Matsuda index and the 1/homeostasis model assessment of insulin resistance were calculated as SI. In each group, SI was estimated by using eight ML methods, based only on physical indicators, as well as by using physical indicators together with lipid and fasting glucose levels. Moreover, 11 lipid-related estimates for SI were calculated.

**Results:**

Estimates by extreme gradient boosting showed the best correlations with SI indices among eight ML methods. According to feature importance and SHapley Additive exPlanations values, the contribution of each clinical factor to SI differed greatly by age and glucose tolerance status. Relationships of lipid-related estimates with SI were weaker than those of ML-derived estimates.

**Conclusions:**

It was possible to estimate SI using ML based only on physical indicators, or those with lipid and fasting glucose levels. The results also imply that it would be difficult to establish universal and robust estimates for SI using conventional parameters. Further validation studies are necessary in diverse ethnic groups with various body composition.

## Introduction

Type 2 diabetes mellitus, which accounts for approximately 90% of all patients with diabetes mellitus, develops mainly due to insufficient sensitivity to insulin (1). As a risk factor for insufficient sensitivity to insulin, the importance of metabolic disorders such as obesity, especially abdominal obesity, hypertension, and dyslipidemia has been established. Metabolic disorders are also reported to be associated with health problems such as cancer and cardiovascular disease (2). The gold standards for estimating insulin sensitivity (SI) are the glucose clamp method and the intravenous glucose tolerance test with minimal model analysis, but these are laborious and not suitable for epidemiological studies (3). Both the homeostasis model assessment of insulin resistance (HOMA-IR) and Matsuda index (ISI-Matsuda) allow SI to be easily assessed. It has been reported that HOMA-IR strongly reflects hepatic SI (4, 5), whereas ISI-Matsuda strongly reflects whole-body SI (6, 7). It is important to calculate a formula that correlates strongly with these SI indices using conventional clinical parameters.

In addition to body mass index (BMI), waist circumference (WC), WC/hip circumference ratio, and WC/height (Ht) ratio as conventional means of assessing health problems due to obesity with decreased SI, the body shape index and body roundness index (BRI) have also been proposed (8, 9), and we have also reported a correlation between BRI and SI (10). In addition to physical indicators, several methods of estimating SI from simple indicators have also been reported, including those based on lipid and fasting glucose levels, such as triglycerides/high density lipoprotein (TG/HDL) (11), lipid accumulation product (LAP) (12), visceral adiposity index (VAI) (13), dysfunctional adiposity index (DAI) (14), triglyceride glucose index (TyG index) (15), the product of TyG index × BMI, etc. (16, 17), atherogenic index of plasma (AIP) (18), metabolic score for insulin resistance (METS-IR) (19), and waist-triglyceride index (WTI) (20). Some of these indicators for SI estimation have been established on a theoretical basis, but others have been set arbitrarily, and the correlations between these indicators and SI are not always robust.

The use of machine learning (ML) has attracted attention as a way of overcoming these weaknesses. Recently, ML has been used to create prediction equations that achieve a strong correlation between SI and physical indicators such as BMI and blood pressure (BP) as the component factors of metabolic syndrome, in addition to lipid and fasting glucose levels (21–23). Park et al. (2022) developed a model in a Korean population-based cohort using HOMA-IR as the outcome measure, Tsai et al. (2023) used data from the US National Health and Nutrition Examination Survey and a Taiwan cohort of adults without diabetes, also focusing on HOMA-IR, and Zhang et al. (2024) developed a machine learning-augmented algorithm in Chinese community and primary care populations. In these previous studies, only HOMA-IR, which is thought to mainly reflect hepatic SI, was used, whereas ISI-Matsuda, which is thought to reflect whole-body SI, was not investigated. The ability to predict SI using only physical indicators such as BMI and BP was also not investigated. Furthermore, age, blood glucose, and lipid levels in these studies were non-uniform, and there was a lack of clarity regarding subject characteristics such as the proportion of subjects with glucose intolerance and lipid disorders, and details of the drugs used to treat those disorders. Various ML methods were used in these studies, but Park et al. and Tsai et al. stated that extreme gradient boosting (XGBoost) was useful among seven and four ML methods tested in their studies (21, 22). Zhang et al. reported that LightGBM was the best ML method for predicting SI among eight ML methods tested, but XGBoost also showed very similar results to LightGBM in their study (23). XGBoost and LightGBM may capture complex non-linear relationships with higher accuracy than other ML methods and be well suited for handling tabular data. XGBoost grows trees evenly to reduce overfitting and ensure stability, whereas LightGBM grows loss-reducing branches for faster, more accurate learning on large datasets, although with greater overfitting risk. Apart from SI estimation, several ML studies have reported attempts to identify risk factors for diabetes and for diabetes combined with cardiovascular diseases using SHapley Additive exPlanations (SHAP) and feature importance analyses (24–26). In these reports, SHAP and feature importance analyses could reveal each risk factor with high predictive accuracy.

We hypothesized that SI can be estimated using ML based on physical indicators only and by physical indicators together with parameters such as lipid and fasting glucose levels. This hypothesis was tested in cohorts of young and middle-aged Japanese men and women who underwent a 75-g oral glucose tolerance test (OGTT) and whose glucose tolerance was precisely assessed. From the 75-g OGTT, both HOMA-IR and ISI-Matsuda were calculated as indicators of SI. The ability to estimate SI by ML was investigated for HOMA-IR and ISI-Matsuda using only physical indicators or using lipid and fasting glucose levels in addition to physical indicators. SHAP and feature importance analyses were also adopted to reveal factors contributing to SI in the cohorts.

## Materials and methods

### Participants

The study participants were 1,268 medical students at Jichi Medical University, Tochigi, Japan (age <40 years) who had normal glucose tolerance (NGT), from among approximately 1,400 students who had undergone a 75-g OGTT between December 2002 and April 2015 (Jichi cohort). NGT was defined based on Japan Diabetes Society criteria (fasting plasma glucose [PG] <110 mg/dL and 120-min value <140 mg/dL) (27). Subjects with triglyceride (TG) levels >400 mg/dL were excluded because of the use of the Friedewald formula described below. The study in the Jichi cohort was approved by the ethics committee of Jichi Medical University (approval no. EKI 09-45). Written, informed consent was obtained from all participants after providing them with complete information on the purposes of the study.

Data from health examinees, aged 30–65 years, at Hokuriku Central Hospital, Toyama, Japan, were also analyzed (Hokuriku cohort). The detailed characteristics of the study population have been described elsewhere (28, 29). Briefly, 1,723 participants who visited the hospital between April 2006 and March 2010 were enrolled in this study after excluding those who had hemoglobin A1c values ≥6.5% and TG >400 mg/dL, who had a known history of diabetes mellitus and/or were taking antidiabetic agents, who were taking antihypertensive and lipid-lowering agents, who had undergone gastrectomy, or who were taking steroids or anticancer drugs. All participants were divided into NGT and glucose intolerance (GI) groups. GI included both newly diagnosed diabetes mellitus, defined based on the above criteria (fasting PG ≥126 mg/dL and/or 120-min value ≥200 mg/dL) (27), and non-diabetic hyperglycemia. The study in the Hokuriku cohort was approved by the ethics committee at Hokuriku Central Hospital. Written, informed consent was obtained from all participants after providing complete information on the purposes of the study.

### Measurements and calculation of SI

PG concentrations were measured using a glucose oxidase assay, and insulin levels were measured using an immunoradiometric assay for immunoreactive insulin (IRI) (Insulin RIA Beads II; Yamasa, Tokyo, Japan), as described previously (Jichi cohort) (30). Serum IRI concentrations were determined using a chemiluminescence immunoassay (Siemens Healthcare Diagnostics, Tokyo, Japan) at a commercial laboratory (BML, Inc., Tokyo, Japan) (Hokuriku cohort) (28, 29). The antibodies used in both insulin assays did not cross-react with proinsulin. In the 75-g OGTT, PG and IRI levels were measured under fasting conditions (preloading) and 120 min after glucose loading; these are abbreviated as PG0 and PG120, and IRI0 and IRI120, respectively.

Similar to our previous studies (30, 31), the following parameters were used. Whole-body SI as determined by ISI-Matsuda was calculated as: ISI-Matsuda = 10,000/[sqrt (PG0 × PG120 × IRI0 × IRI120)] (6, 32). In addition, 1/HOMA-IR was used primarily as a measure of hepatic SI. HOMA-IR was calculated as [PG0 × IRI0/405] (4). The units for PG and IRI were milligrams per deciliter and microunits per milliliter, respectively, for calculating ISI-Matsuda and HOMA-IR.

The quintile for ISI-Matsuda and 1/HOMA-IR in the NGT of each cohort was adopted as the cutoff for decreased SI, i.e., insulin resistance. The quintile was adopted according to the previous study (33). In the Jichi cohort, an ISI-Matsuda ≤5.6 and a 1/HOMA-IR ≤0.517 were used. In the Hokuriku cohort, an ISI-Matsuda ≤6.1 and a 1/HOMA-IR ≤0.728 were used.

### Questionnaires and measurements of background factors

Data on age and sex were obtained through questionnaires. High-density lipoprotein cholesterol (HDL), TG, and total cholesterol (T-chol) levels were measured in serum collected under fasting conditions. The units for HDL, TG, and T-chol were milligrams per deciliter. The low-density lipoprotein cholesterol (LDL) concentration was calculated using the Friedewald formula (LDL = T-chol − HDL − TG/5) (34). Systolic and diastolic blood pressures (SBP and DBP) were measured after the participant had been seated at rest for 5 min. Mean blood pressure (MBP) was calculated as DBP + (SBP − DBP)/3. BMI was calculated as the weight in kilograms divided by Ht in meters squared. WC was measured at the umbilical level with the subject standing (35). The WC/Ht ratio was also calculated.

### Estimates by machine learning for SI

Prediction equations using eight ML methods were created to predict SI. Multiple regression analysis (MRA), a neural network (ANN), decision tree (DT), random forest (RF), boosting tree (BT), K nearest neighbor (KNN), support vector machine (SVM), and extreme gradient boosting (XGBoost) were used as ML methods. Seven factors that were measured in both cohorts and had an established association with SI were used as predictors of SI. First, the three physical indicators BMI, WC/Ht ratio, and MBP were used as input factors in each ML method, and the prediction equations for ISI-Matsuda and 1/HOMA-IR were calculated. The WC/Ht ratio was chosen as the physical indicator for input because, in our previous report, the WC/Ht ratio had a higher correlation with SI than WC (10). Lipid and fasting glucose levels were then added to the three factors, and these seven factors (BMI, WC/Ht ratio, MBP, HDL, TG, LDL, and PG0) were used as input factors in each ML method, and prediction equations for SI were calculated. In the ML methods that showed the best correlation with SI, sex was also entered, giving a total of eight factors, and the feature importance and SHAP values of these factors were calculated. Feature importance provided global insights, whereas SHAP clarified positive or negative impact of each factor at the individual level.

### Measurements of lipid-related estimates for SI

Similar to previous studies, the following seven estimates were calculated.

LAP^12^: Men: [WC (cm) − 65] × [TG (mmol/l)]; Women: [WC (cm) − 58] × [TG (mmol/l)].

VAI^13^: Men: [WC/(39.68 + 1.88 × BMI)] × (TG/1.03) × (1.31/HDL); Women: [WC/(36.58 + 1.89 × BMI)] × (TG/0.81) × (1.52/HDL), where both TG and HDL levels are expressed in mmol/l.

DAI^14^: Men: [WC/(22.79 + 2.68 × BMI)] × (TG/1.37) × (1.19/HDL); Women: [WC/(24.02 + 2.37 × BMI)] × (TG/1.32) × (1.43/HDL), where both TG and HDL levels are expressed in mmol/l.

TYG index^15^: Ln [TG (mg/dl) × PG0 (mg/dl)/2].

AIP^18^: log [TG (mmol/l)/HDL (mmol/l)].

METS-IR^19^: Ln [2 × PG0 (mg/dl) + TG (mg/dl)] × BMI/Ln [HDL (mg/dl)].

WTI^20^: Ln [TG (mg/dL) × WC (cm)/2].

### Statistical analysis

JMP Pro (version 17, SAS Institute Inc., Cary, NC, USA) was used for all statistical analyses except for the receiver-operating characteristic (ROC) curve analysis. Missing values were not included in the analysis. In this study, the default settings of the predictive modeling platform were utilized in all ML algorithms. It has been reported that ML analyses conducted with default settings generally achieve high accuracy (36–38), and the same approach was adopted in this study. The details of the default settings are described in the **Supplementary Table 1**. Since almost none of the variables had a normal distribution, results are expressed as median (25th percentile, 75th percentile) values.

The correlations of ML-derived and lipid-related estimates with SI were tested using Spearman’s rank-correlation coefficients on bivariate analysis. The correlations of ML-derived estimates with SI were also evaluated by calculating coefficient of determination (R²), root mean squared error (RMSE), and mean absolute error (MAE) and were also shown as calibration plots.

ROC curves and the areas under the ROC curves (AUCs) were used to assess the ability of the best estimates to detect insulin resistance, using EZR ver. 1.61 (Saitama Medical Center, Jichi Medical University, Saitama, Japan) (39). If the lower limit of the 95% confidence interval (CI) for the AUC was below 0.50, that index was considered to not have the ability to detect insulin resistance. Optimal cutoff values were determined by maximization of the Youden index (sensitivity + specificity − 1). The Brier score was also calculated. For all statistical tests, values of *P* < 0.05 were considered significant.

## Results

### Characteristics of the entire cohort

The characteristics of the study participants stratified by sex are shown in **Table 1**. The Jichi cohort (n = 1,268) included only persons with NGT and was a young cohort with few cases of obesity, hypertension, and dyslipidemia. The participants in the Hokuriku cohort were sorted into an NGT-only group (n = 1,276) and a group with GI (n = 447). Both groups in the Hokuriku cohort consisted of middle-aged persons who had higher BMI, WC, WC/Ht ratio, BP, lipids, glucose, and lipid-related estimates than the young persons with NGT (the Jichi cohort). The Hokuriku cohort included 447 persons with GI (non-diabetic hyperglycemia, n = 392; newly diagnosed diabetes mellitus, n = 55), accounting for 26% of the total cohort. The group with GI in the Hokuriku cohort did not appear to have any major differences in age, BMI, WC, height, WC/Ht ratio, BP, lipids, or lipid-related estimates compared with the NGT group of the same cohort; however, their glucose levels (PG0 and PG120) were higher, and their ISI-Matsuda was lower.

**Table 1:** Characteristics of the participants in the Jichi and Hokuriku cohorts.

	Jichi cohort NGT (young)		Hokuriku cohort NGT (middle-aged)		Hokuriku cohort GI (middle-aged)	
	Male (n=977)	Female (n=291)	Male (n=837)	Female (n=439)	Male (n=322)	Female (n=125)
Age (y)	23 (22, 23)	23 (22, 24)	51 (45, 57)	55 (50, 59)	54 (48, 59)	57 (50, 59)
BMI (kg/m^2^)	21.7 (20.5, 23.3)	20.0 (18.8, 21.1)	23.7 (22.1, 25.4)	22.2 (20.5, 24.0)	24.3 (22.4, 26.3)	23.0 (20.9, 25.2)
WC (cm)	77 (73, 81)	68 (64, 72)	83 (79, 88)	80 (74, 85)	86 (81, 91)	83 (75, 90)
Height (cm)	172 (169, 176)	160 (156, 163)	170 (166, 174)	157 (153, 160)	170 (166, 174)	157 (154, 160)
WC/Ht ratio	0.44 (0.42, 0.47)	0.43 (0.41, 0.45)	0.49 (0.46, 0.52)	0.51 (0.47, 0.54)	0.50 (0.48, 0.53)	0.53 (0.47, 0.58)
SBP (mmHg)	120 (115, 128)	108 (103, 115)	127 (116, 139)	119 (109, 132)	134 (122, 146)	126 (113, 141)
DBP (mmHg)	67 (63, 73)	63 (60, 67)	79 (73, 87)	73 (67, 81)	84 (76, 92)	78 (71, 86)
MBP (mmHg)	85 (80, 91)	84 (79, 88)	95 (88, 104)	88 (81, 98)	101 (92, 110)	94 (85, 105)
HDL (mg/dL)	59 (52, 67)	68 (59, 77)	55 (47, 64)	64 (56, 75)	55 (47, 66)	65 (55, 74)
TG (mg/dL)	63 (49, 86)	52 (40, 67)	111 (80, 153)	81 (62, 113)	125 (89, 174)	85 (64, 121)
LDL (mg/dL)	90 (76, 105)	96 (81, 114)	127 (108, 146)	131 (110, 154)	131 (112, 153)	137 (113, 159)
TC (mg/dL)	165 (149, 182)	176 (154, 197)	209 (186, 230)	218 (193, 241)	217 (195, 241)	224 (203, 246)
PG0 (mg/dL)	88 (82, 94)	85 (80, 91)	96 (91, 101)	93 (88, 97)	110 (100, 115)	102 (95, 109)
PG120 (mg/dL)	91 (78, 103)	94 (80, 107)	106 (93, 120)	105 (92, 116)	152 (140, 171)	156 (143, 173)
IRI0 (μU/mL)	5.8 (4.3, 8.2)	6.1 (4.6. 8.5)	3.7 (2.7, 5.0)	3.5 (2.8, 4.8)	4.0 (2.9, 5.7)	4.0 (2.9, 5.8)
IRI120 (μU/mL)	23.2 (14.0, 37.6)	39.0 (25.5, 58.2)	20.4 (13.0, 32.0)	21.4 (15.0, 31.8)	33.2 (20.2, 57.5)	38.5 (26.2, 54.4)
HOMA-IR	1.26 (0.90, 1.77)	1.30 (0.94, 1.80)	0.86 (0.64, 1.19)	0.83 (0.64, 1.11)	1.08 (0.77, 1.53)	0.99 (0.73, 1.51)
1/HOMA-IR	0.79 (0.56, 1.11)	0.77 (0.55, 1.06)	1.16 (0.84, 1.57)	1.20 (0.90, 1.57)	0.92 (0.65, 0.92)	1.01 (0.66, 1.37)
ISI-Matsuda	10.1 (6.4, 14.6)	7.4 (5.3, 10.0)	11.7 (8.0, 17.5)	11.6 (8.3, 16.5)	7.1 (4.5, 10.6)	6.2 (4.5, 9.0)
TG/HDL	1.08 (0.77, 1.55)	0.76 (0.58, 1.06)	2.02 (1.31, 3.06)	1.24 (0.85, 1.93)	2.21 (1.50, 3.56)	1.43 (0.92, 2.53)
LAP	8.0 (4.6, 13.8)	5.7 (3.4, 9.0)	22.0 (13.3, 35.6)	19.6 (11.5, 30.5)	28.3 (17.0, 48.0)	26.3 (12.9, 48.5)
VAI	0.56 (0.40, 0.83)	0.57 (0.43, 0.81)	1.10 (0.72, 1.73)	1.04 (0.69, 1.58)	1.21 (0.79, 2.05)	1.19 (0.74, 2.07)
DAI	0.38 (0.27, 0.56)	0.34 (0.26, 0.49)	0.74 (0.48, 1.14)	0.62 (0.41, 0.93)	0.81 (0.54, 1.35)	0.71 (0.45, 1.19)
TYG	7.92 (7.64, 8.25)	7.66 (7.44, 7.96)	8.57 (8.25, 8.90)	8.24 (7.96, 8.58)	8.83 (8.49, 9.13)	8.49 (8.12, 8.92)
TYG×BMI	172 (160, 189)	154 (143, 165)	201 (185, 222)	182 (165, 202)	215 (192, 239)	196 (174, 225)
TYG×WC	608 (564, 657)	523 (489, 562)	716 (657, 771)	654 (588, 718)	757 (693, 818)	703 (611, 784)
TYG×WC/Ht	3.53 (3.28, 3.82)	3.29 (3.07, 3.54)	4.20 (3.88, 4.54)	4.18 (3.75, 4.60)	4.46 (4.11, 4.81)	4.54 (3.87, 5.01)
AIP	−0.33 (−0.48, −0.17)	−0.48 (−0.60, −0.34)	−0.054 (−0.24, 0.13)	−0.27 (−0.43, −0.074)	−0.015 (−0.18, 0.19)	−0.20 (−0.40, 0.043)
METS-IR	29.3 (27.2, 32.2)	25.5 (23.6, 27.5)	33.8 (30.5, 37.5)	29.8 (26.9, 33.4)	35.9 (31.7, 40.2)	31.6 (28.2, 37.0)
WTI	7.79 (7.50, 8.11)	7.48 (7.21, 7.73)	8.42 (8.10, 8.78)	8.07 (7.77, 8.44)	8.59 (8.21, 8.95)	8.24 (7.87, 8.72)

Date are shown as median (25th percentile, 75th percentile) values.

NGT, normal glucose tolerance; GI, glucose intolerance; BMI, body mass index; WC, waist circumference; Ht, height; SBP, systolic blood pressure; DBP, diastolic blood pressure; MBP, mean blood pressure; HDL, high-density lipoprotein cholesterol; TG, triglycerides; LDL, low-density lipoprotein cholesterol; PG, plasma glucose; IRI, immunoreactive insulin; HOMA-IR, homeostasis model assessment of insulin resistance; ISI-Matsuda, Matsuda index; LAP, lipid accumulation product; VAI, visceral adiposity index; DAI, dysfunctional adiposity index; TYG, triglycerides and glucose index; AIP, atherogenic index of plasma; METS-IR, metabolic score for insulin resistance; WTI, waist-triglyceride index.

### In the ML methods, XGBoost-derived estimates had the best relationship with SI in each cohort

The correlations between SI (1/HOMA-IR and ISI-Matsuda) and ML-derived estimates using three factors are shown in **Table 2**, and the correlations between SI and ML-derived estimates using seven factors are shown in **Table 3**. Of the ML methods with three factors, XGBoost-derived estimates showed the best correlation with SI in all subgroups (Spearman’s ρ= 0.81–1.00), followed by RF-derived estimates (Spearman’s ρ= 0.68–0.85). There were no differences between the correlation of XGBoost-derived estimates with 1/HOMA-IR and ISI-Matsuda (**Table 2**). Very similar results were seen for ML methods with seven factors, but Spearman’s ρ values in all subgroups were higher than for ML methods with three factors (Spearman’s ρ = 0.87–1.00, **Tables 2**, **3**). Spearman’s ρ values for SI were slightly better for NGT in the middle-aged group than in the young group (Hokuriku cohort vs. Jichi cohort NGT) and higher in the Hokuriku cohort GI than for NGT in both cohorts (**Tables 2**, **3**).

**Table 2:** Non-parametric Spearman rank correlation coefficients of machine learning indices by three factors for 1/HOMA-IR and ISI-Matsuda by sex.

	For 1/HOMA-IR				For ISI-Matsuda			
	Male		Female		Male		Female	
	ρ	*P*	ρ	*P*	ρ	*P*	ρ	*P*
Jichi cohort NGT (young)								
MRA	0.32	<0.0001	0.27	<0.0001	0.33	<0.0001	0.27	<0.0001
ANN	0.32	<0.0001	0.28	<0.0001	0.33	<0.0001	0.26	<0.0001
DT	0.69	<0.0001	0.60	<0.0001	0.71	<0.0001	0.63	<0.0001
RF	0.77	<0.0001	0.77	<0.0001	0.79	<0.0001	0.77	<0.0001
BT	0.72	<0.0001	0.56	<0.0001	0.77	<0.0001	0.60	<0.0001
KNN	0.37	<0.0001	0.33	<0.0001	0.43	<0.0001	0.34	<0.0001
SVM	0.36	<0.0001	0.38	<0.0001	0.38	<0.0001	0.39	<0.0001
XGBoost	0.87	<0.0001	0.97	<0.0001	0.84	<0.0001	0.98	<0.0001
Hokuriku cohort NGT (middle-aged)								
MRA	0.49	<0.0001	0.44	<0.0001	0.47	<0.0001	0.46	<0.0001
ANN	0.46	<0.0001	0.42	<0.0001	0.47	<0.0001	0.43	<0.0001
DT	0.74	<0.0001	0.71	<0.0001	0.73	<0.0001	0.76	<0.0001
RF	0.74	<0.0001	0.72	<0.0001	0.78	<0.0001	0.82	<0.0001
BT	0.60	<0.0001	0.50	<0.0001	0.67	<0.0001	0.68	<0.0001
KNN	0.51	<0.0001	0.43	<0.0001	0.51	<0.0001	0.50	<0.0001
SVM	0.56	<0.0001	0.52	<0.0001	0.52	<0.0001	0.51	<0.0001
XGBoost	0.81	<0.0001	0.93	<0.0001	0.82	<0.0001	0.95	<0.0001
Hokuriku cohort GI (middle-aged)								
MRA	0.45	<0.0001	0.63	<0.0001	0.50	<0.0001	0.51	<0.0001
ANN	0.45	<0.0001	0.63	<0.0001	0.49	<0.0001	0.51	<0.0001
DT	0.66	<0.0001	0.81	<0.0001	0.72	<0.0001	0.72	<0.0001
RF	0.68	<0.0001	0.85	<0.0001	0.81	<0.0001	0.76	<0.0001
BT	0.49	<0.0001	0.84	<0.0001	0.66	<0.0001	0.77	<0.0001
KNN	0.42	<0.0001	0.67	<0.0001	0.53	<0.0001	0.53	<0.0001
SVM	0.52	<0.0001	0.69	<0.0001	0.53	<0.0001	0.59	<0.0001
XGBoost	0.95	<0.0001	1.00	<0.0001	0.96	<0.0001	1.00	<0.0001

HOMA-IR, homeostasis model assessment of insulin resistance; ISI-Matsuda, Matsuda index; NGT, normal glucose tolerance; GI, glucose intolerance; MRA, multiple regression analysis; ANN, a neural network; DT, decision tree; RF, random forest; BT, boosting tree; KNN, K nearest neighbor; SVM, support vector machine; XGBoost, extreme gradient boosting.

**Table 3:** Non-parametric Spearman rank correlation coefficients of machine learning indices by seven factors for 1/HOMA-IR and ISI-Matsuda by sex.

	For 1/HOMA-IR				For ISI-Matsuda			
	Male		Female		Male		Female	
	ρ	*P*	ρ	*P*	ρ	*P*	ρ	*P*
Jichi cohort NGT (young)								
MRA	0.48	<0.0001	0.44	<0.0001	0.45	<0.0001	0.34	<0.0001
ANN	0.49	<0.0001	0.46	<0.0001	0.46	<0.0001	0.28	<0.0001
DT	0.74	<0.0001	0.75	<0.0001	0.78	<0.0001	0.73	<0.0001
RF	0.87	<0.0001	0.88	<0.0001	0.88	<0.0001	0.89	<0.0001
BT	0.81	<0.0001	0.68	<0.0001	0.86	<0.0001	0.63	<0.0001
KNN	0.54	<0.0001	0.43	<0.0001	0.49	<0.0001	0.41	<0.0001
SVM	0.62	<0.0001	0.70	<0.0001	0.60	<0.0001	0.66	<0.0001
XGBoost	0.90	<0.0001	0.99	<0.0001	0.92	<0.0001	0.99	<0.0001
Hokuriku cohort NGT (middle-aged)								
MRA	0.56	<0.0001	0.52	<0.0001	0.51	<0.0001	0.54	<0.0001
ANN	0.56	<0.0001	0.52	<0.0001	0.54	<0.0001	0.52	<0.0001
DT	0.80	<0.0001	0.75	<0.0001	0.79	<0.0001	0.79	<0.0001
RF	0.81	<0.0001	0.76	<0.0001	0.86	<0.0001	0.89	<0.0001
BT	0.71	<0.0001	0.62	<0.0001	0.77	<0.0001	0.82	<0.0001
KNN	0.59	<0.0001	0.48	<0.0001	0.59	<0.0001	0.61	<0.0001
SVM	0.74	<0.0001	0.74	<0.0001	0.69	<0.0001	0.71	<0.0001
XGBoost	0.87	<0.0001	0.94	<0.0001	0.89	<0.0001	0.98	<0.0001
Hokuriku cohort GI (middle-aged)								
MRA	0.52	<0.0001	0.69	<0.0001	0.52	<0.0001	0.57	<0.0001
ANN	0.46	<0.0001	0.69	<0.0001	0.39	<0.0001	0.56	<0.0001
DT	0.79	<0.0001	0.85	<0.0001	0.77	<0.0001	0.82	<0.0001
RF	0.77	<0.0001	0.90	<0.0001	0.86	<0.0001	0.87	<0.0001
BT	0.58	<0.0001	0.90	<0.0001	0.75	<0.0001	0.83	<0.0001
KNN	0.47	<0.0001	0.72	<0.0001	0.52	<0.0001	0.42	<0.0001
SVM	0.79	<0.0001	0.82	<0.0001	0.69	<0.0001	0.82	<0.0001
XGBoost	0.98	<0.0001	1.00	<0.0001	0.99	<0.0001	1.00	<0.0001

HOMA-IR, homeostasis model assessment of insulin resistance; ISI-Matsuda, Matsuda index; NGT, normal glucose tolerance; GI, glucose intolerance; MRA, multiple regression analysis; ANN, a neural network; DT, decision tree; RF, random forest; BT, boosting tree; KNN, K nearest neighbor; SVM, support vector machine; XGBoost, extreme gradient boosting.

R², RMSE, and MAE are shown in **Supplementary Table 2** (by using three factors) and in **Supplementary Table 3** (by using seven factors). XGBoost-derived estimates for 1/HOMA-IR and ISI-Matsuda showed the highest R^2^ and the lowest RMSE and MAE in all subgroups. R², RMSE, and MAE with seven factors were slightly better than those with three factors.

The calibration plots are shown in **Supplementary Figure 1** (by using three factors) and in **Supplementary Figure 2** (by using seven factors). XGBoost-derived estimates with three or seven factors showed strong linear associations with the actual values of 1/HOMA-IR and ISI-Matsuda in all subgroups.

### The ROC analyses with XGBoost-derived estimates using seven factors showed good AUCs for detecting insulin resistance

The results of the ROC analyses with XGBoost-derived estimates using seven factors for the presence or absence of insulin resistance based on 1/HOMA-IR and ISI-Matsuda are shown in **Table 4**. The AUCs were significant for the ability of the XGBoost-derived estimates in all subgroups to identify insulin resistance. The XGBoost-derived estimates showed good AUCs, sensitivity, and specificity in all subgroups. The AUC in men with NGT of the Hokuriku cohort was the lowest (0.922), whereas the AUCs were high in women overall (0.972–1.000) and in men with GI of the Hokuriku cohort (0.986–0.996). The Brier scores were consistent with the AUC results.

**Table 4:** Area under the curve, cutoff values, sensitivity, specificity, and Brier scores of XGBoost predictors by seven factors for the presence of insulin resistance in both sexes.

	Male					Female				
	AUC (95%CI)	Cutoff value	Sensitivity (%)	Specificity (%)	Brier score	AUC (95%CI)	Cutoff value	Sensitivity (%)	Specificity (%)	Brier score
Jichi cohort NGT (young)										
For the presence of insulin resistance by 1/HOMA-IR	0.935 (0.917-0.953)	0.696	82	89	0.0199	0.993 (0.987-0.999)	0.584	98	94	0.000736
For the presence of insulin resistance by ISI-Matsuda	0.946 (0.930-0.961)	8.360	90	84	0.0113	0.997 (0.993-1.000)	5.724	95	100	0.00217
Hokuriku cohort NGT (middle-aged)										
For the presence of insulin resistance by 1/HOMA-IR	0.922 (0.898-0.946)	0.996	87	81	0.0123	0.972 (0.957-0.987)	0.992	97	85	0.000796
For the presence of insulin resistance by ISI-Matsuda	0.935 (0.917-0.954)	10.123	93	79	0.00651	0.985 (0.973-0.997)	7.651	97	92	0.000289
Hokuriku cohort GI (middle-aged)										
For the presence of insulin resistance by 1/HOMA-IR	0.986 (0.976-0.995)	0.815	97	93	0.00277	1.000 (1.000-1.000)	0.713	100	100	0.00000693
For the presence of insulin resistance by ISI-Matsuda	0.996 (0.992-0.999)	6.149	96	97	0.00358	1.000 (1.000-1.000)	6.040	100	100	0.0000645

XGBoost, extreme gradient boosting; HOMA-IR, homeostasis model assessment of insulin resistance; ISI-Matsuda, Matsuda index; NGT, normal glucose tolerance; GI, glucose intolerance; AUC, area under the receiver operating characteristic curve; CI, confidence interval.

### The feature importance revealed that the factors showing a high contribution to SI differed greatly by age and glucose tolerance status

**Figure 1:**
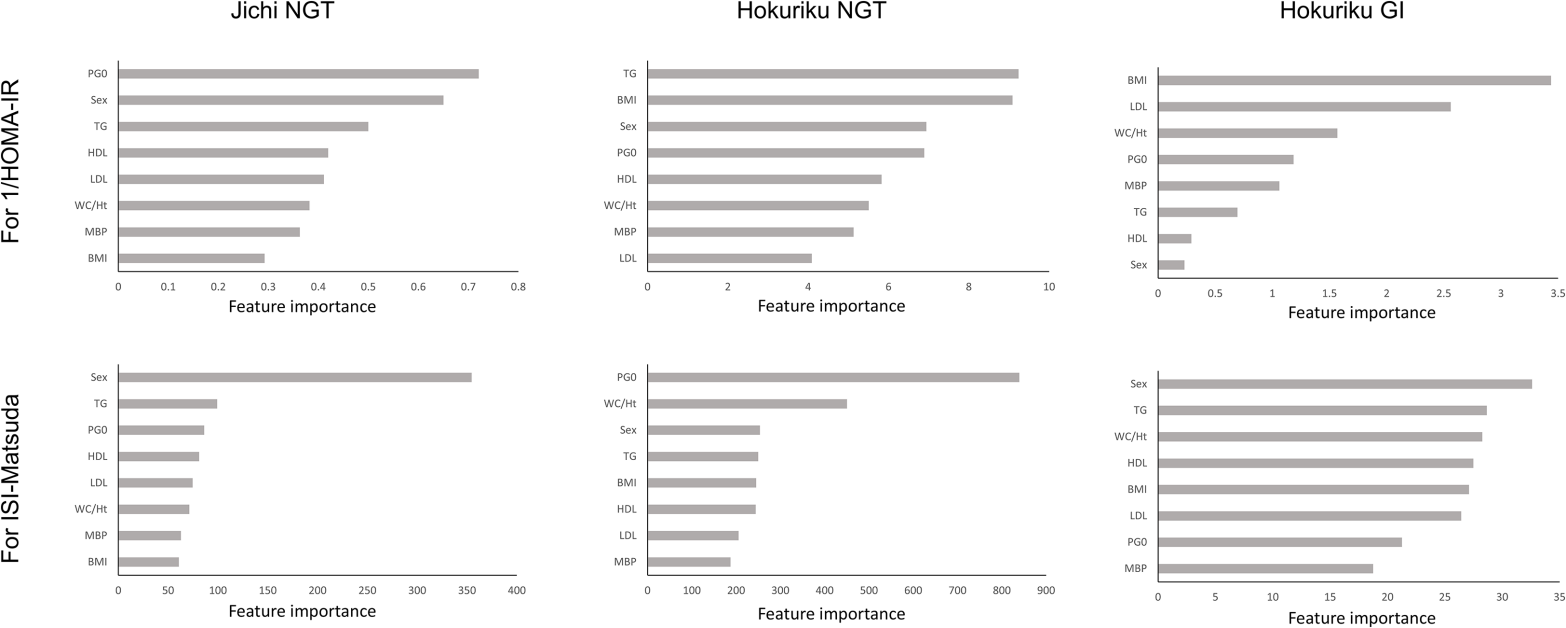
Feature importance for 1/HOMA-IR and ISI-Matsuda by XGBoost in the Jichi cohort and in normal glucose tolerance (NGT) and glucose intolerance (GI) in the Hokuriku cohort. HOMA-IR, homeostasis model assessment of insulin resistance; ISI-Matsuda, Matsuda index; BMI, body mass index; WC, waist circumference; Ht, height; MBP, mean blood pressure; HDL, high-density lipoprotein cholesterol; TG, triglycerides; LDL, low-density lipoprotein cholesterol; PG0, fasting plasma glucose.

For both sexes in both groups, there was a good correlation between SI and XGBoost-derived estimates using seven factors, and good AUC for detecting insulin resistance. Feature importance was therefore calculated for the NGT and GI groups in the Jichi cohort and the Hokuriku cohort by using eight input factors (the seven factors and sex) with XGBoost ML (**Figure 1**). Sex, WC/Ht ratio, TG, and PG0 showed a high contribution to SI in many groups, but the factors showing a high contribution differed greatly by age and glucose tolerance status.

### The SHAP values also revealed that the factors showing a high contribution to SI differed greatly by age and glucose tolerance status

**Figure 2:**
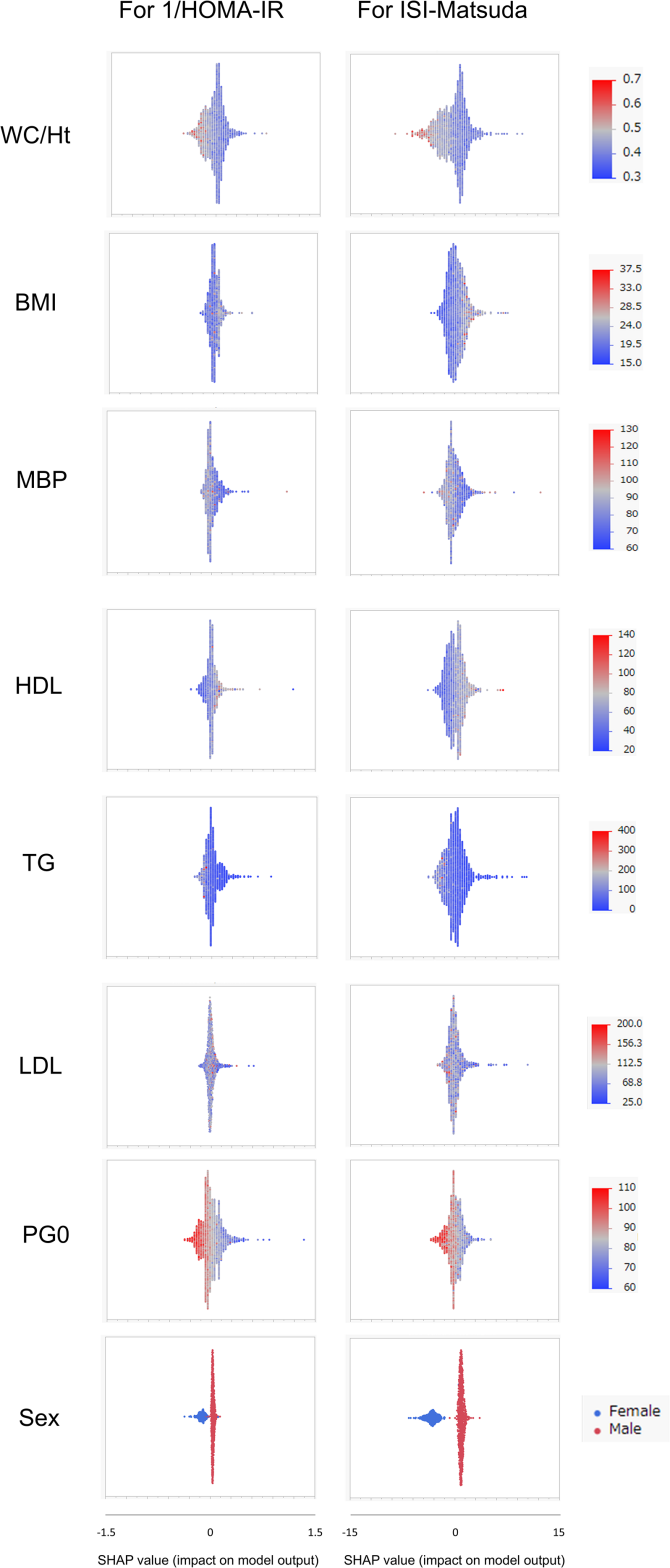
Relative importance of the eight features for insulin sensitivity (SI) prediction, as determined by the extreme gradient boosting (XGBoost) algorithms. Explanation of each feature impact on the SI prediction model by SHAP (Shapley Additive exPlanations) values using XGBoost in the Jichi cohort. HOMA-IR, homeostasis model assessment of insulin resistance; ISI-Matsuda, Matsuda index; WC, waist circumference; Ht, height; BMI, body mass index; MBP, mean blood pressure; HDL, high-density lipoprotein cholesterol; TG, triglycerides; LDL, low-density lipoprotein cholesterol; PG0, fasting plasma glucose.

**Figure 3:**
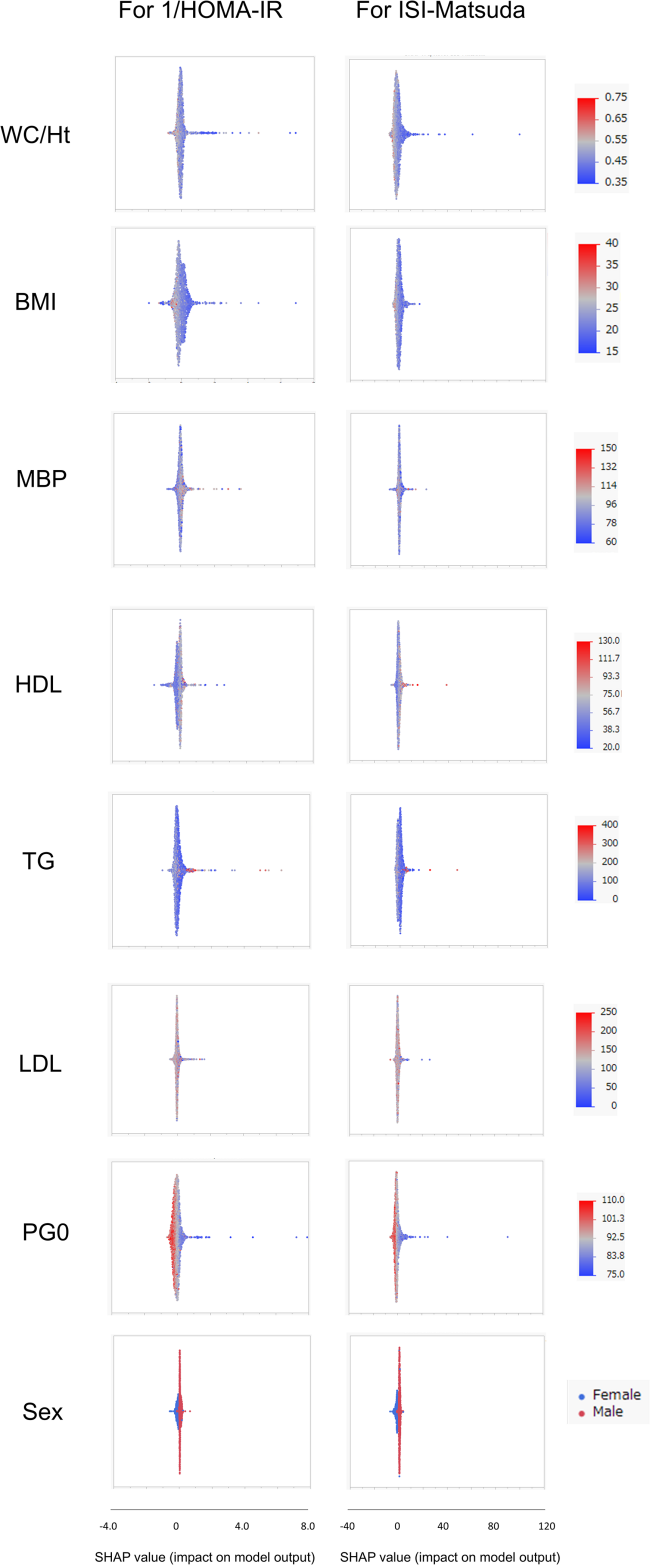
Relative importance of the eight features for insulin sensitivity (SI) prediction, as determined by the extreme gradient boosting (XGBoost) algorithms. Explanation of each feature impact on the SI prediction model by SHAP (Shapley Additive exPlanations) values using XGBoost in the Hokuriku cohort NGT (normal glucose tolerance). HOMA-IR, homeostasis model assessment of insulin resistance; ISI-Matsuda, Matsuda index; WC, waist circumference; Ht, height; BMI, body mass index; MBP, mean blood pressure; HDL, high-density lipoprotein cholesterol; TG, triglycerides; LDL, low-density lipoprotein cholesterol; PG0, fasting plasma glucose.

**Figure 4:**
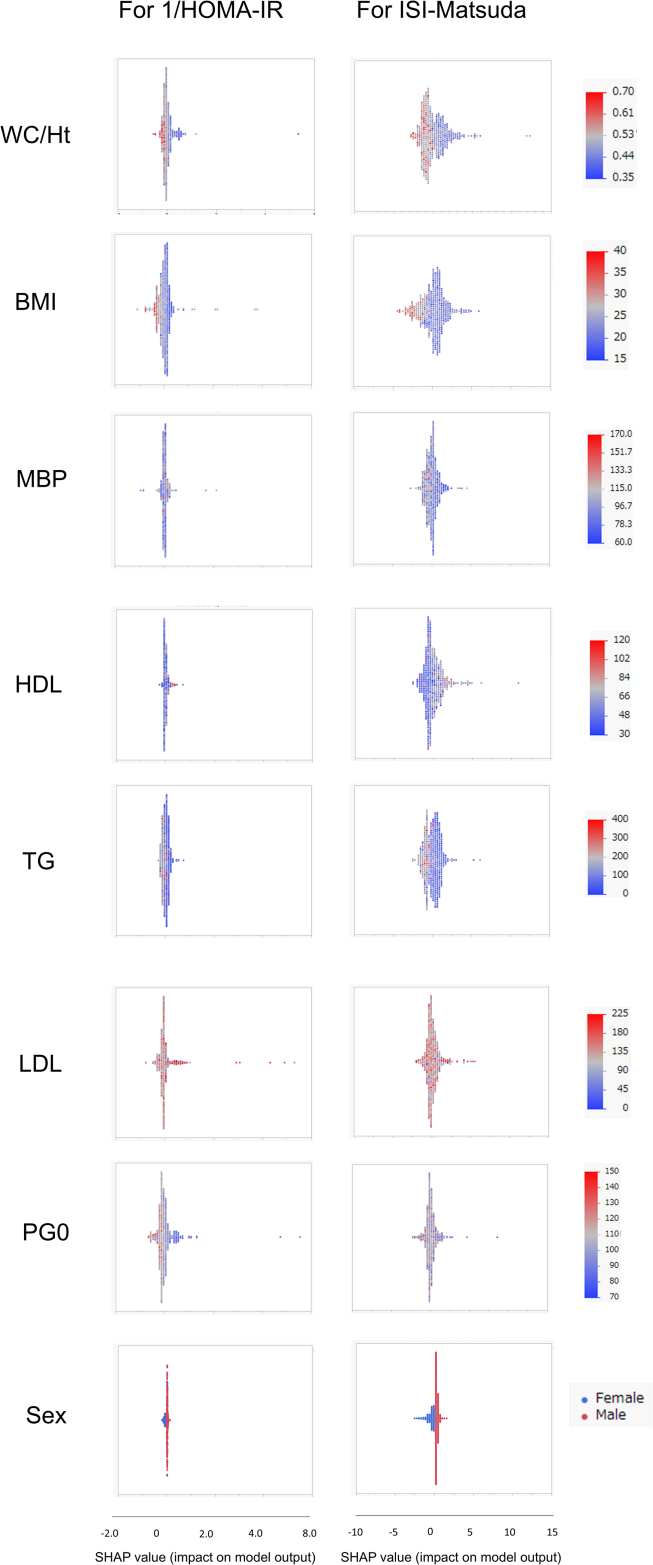
Relative importance of the eight features for insulin sensitivity (SI) prediction, as determined by the extreme gradient boosting (XGBoost) algorithms. Explanation of each feature impact on the SI prediction model by SHAP (Shapley Additive exPlanations) values using XGBoost in the Hokuriku cohort GI (glucose intolerance). HOMA-IR, homeostasis model assessment of insulin resistance; ISI-Matsuda, Matsuda index; WC, waist circumference; Ht, height; BMI, body mass index; MBP, mean blood pressure; HDL, high-density lipoprotein cholesterol; TG, triglycerides; LDL, low-density lipoprotein cholesterol; PG0, fasting plasma glucose.

SHAP values were similarly calculated for the NGT and GI groups in the Jichi cohort and the Hokuriku cohort by using eight input factors (the seven factors and sex) with XGBoost ML. In the Jichi cohort, a positive or negative impact and a significant contribution to the SI prediction equation were seen with WC/Ht ratio, PG0, and sex for 1/HOMA-IR, and with WC/Ht ratio, BMI, TG, PG0, and sex for ISI-Matsuda (**Figure 2**). In the Hokuriku cohort NGT, a positive or negative impact and a significant contribution were seen with BMI, PG0, and sex for 1/HOMA-IR, and with WC/Ht ratio, BMI, TG, PG0, and sex for ISI-Matsuda (**Figure 3**). In the Hokuriku cohort GI, a positive or negative impact and a significant contribution were seen with WC/Ht ratio, BMI, PG0, and sex for 1/HOMA-IR, and with WC/Ht ratio, BMI, and sex for ISI-Matsuda (**Figure 4**). Male sex displayed positive impact, whereas WC/Ht ratio, BMI, and PG0 negative impact in many of the groups. As with feature importance, the factors with a high contribution differed greatly by age and glucose tolerance status.

### Relationships of lipid-related estimates with SI were weaker than for ML-derived estimates

The correlations between lipid-related estimates and SI are shown for each subgroup (**Supplementary Table 4**). Overall, Spearman’s ρ values for SI were lower for lipid-related estimates than for ML-derived estimates (**Tables 2**, **3**). Spearman’s ρ values for SI were higher in the middle-aged group than in the young group, and higher in GI than in NGT. In NGT, Spearman’s ρ values for SI were higher in men than women, and in GI, they were higher in women than men. The product of TyG index and physical indicators, as well as METS-IR, showed relatively strong correlations in all subgroups.

## Discussion

In this study, correlations between SI and ML-derived estimates were calculated using a total of seven factors: the three physical indicators BMI, WC/Ht ratio, and MBP, plus lipid and fasting glucose levels (HDL, TG, LDL, and PG0). In ML-derived estimates using three and seven factors, the prediction equations for XGBoost-derived estimates showed the strongest correlation with SI in all subgroups. XGBoost-derived estimates using seven factors had a stronger association with SI than did those with three factors, but the improvement in correlation with seven factors was moderate. XGBoost-derived estimates using seven factors showed high sensitivity and specificity for detecting insulin resistance. In terms of feature importance in the XGBoost prediction equations, sex, WC/Ht ratio, TG, and PG0 showed a high contribution to SI in many groups. Analysis by SHAP values showed that male sex displayed positive impact, whereas WC/Ht ratio, BMI, and PG0 negative impact on SI. On analyses by feature importance and SHAP values, the contribution of each clinical factor to SI differed greatly by age and glucose tolerance status.

We have previously reported that the physical indicator WC/Ht ratio is strongly correlated with SI, especially in the middle-aged Hokuriku cohort (10), and in the present study, we initially analyzed the relationship between SI and ML-derived estimates using only the physical indicators BMI, WC/Ht ratio, and MBP. Previous studies have shown that physical indicators are an important factor in ML for predicting SI (21–23), but none of those studies reported a relationship between SI and ML-derived estimates when inputs were restricted to physical indicators. Those analyses were also limited to 1/HOMA-IR as the SI index. The present study found that XGBoost-derived estimates using only physical indicators were strongly correlated with both the 1/HOMA-IR and ISI-Matsuda indices of SI (Spearman’s ρ= 0.81–1.00). The results of R², RMSE, and MAE, and calibration plots supported this finding. It is a new finding of this study that the SI of an individual can be estimated with high accuracy by ML using only physical indicators. Previous research has also shown that the AUC of insulin resistance estimated by ML deteriorates very little when the ML input factors are reduced (23). XGBoost-derived estimates using lipid and fasting glucose levels in addition to physical indicators showed a slightly stronger correlation with SI than when using physical indicators alone (Spearman’s ρ= 0.87–1.00). The addition of conventional biochemical indices improved the accuracy of SI estimates, but the effect of this addition was moderate. Previous research has shown that the addition of biochemical indices considerably improves the accuracy of SI estimates (23), but this could be due to differences in the subject populations.

Of the ML methods, XGBoost-derived estimates showed the strongest correlation with SI, consistent with previous reports (21–23). ML performs regression, classification, and clustering from the dataset through iterative training, and it can therefore generate more accurate predictions than traditional statistical methods such as multiple regression analysis (21). Of the ML methods, XGBoost and RF have the highest accuracy because they generate appropriate models by creating numerous decision trees (21). The SI prediction equations produced by XGBoost and RF in the present study were also good (**Tables 2**, **3**). RF performs bagging to reduce overfitting and variance, and it uses independent classifiers. The flaw of RF is that its accuracy does not increase when there is only a small amount of learning data (40). XGBoost performs gradient boosting to reduce bias and variance, uses sequential classifiers, and aggregates predictions of many individually trained classifiers (41). Although XGBoost overfits the data into the model, it can reduce the flaw of RF. Of the ML methods, the XGBoost-derived estimates showed the best correlation with SI. However, in the present study, the ML models were used with default settings, and measures such as overfitting prevention, hyperparameter tuning, and validation had already been implemented by the software vendors. The performance of ML methods other than XGBoost could also improve by fine tuning; therefore, the superiority of XGBoost cannot be insisted.

In the analysis of AUC for detection of insulin resistance, the AUC in XGBoost-derived estimates was high in all groups (0.922–1.000), with good sensitivity (82%–100%) and specificity (79%–100%). The AUC analysis and Brier scores in this study showed that the XGBoost prediction equation can detect insulin resistance with high accuracy. Previous studies have also shown good AUCs in the detection of insulin resistance by ML-derived estimates (21–23). The AUCs from the ROC analysis in the present study were better than those previously reported. This is presumably because AUCs in the present study were calculated within each subgroup stratified by age, sex, and glucose tolerance. In addition, subjects taking antidiabetic, antihypertensive, and lipid-lowering agents were excluded, resulting in analysis of a more homogeneous population than in the previous studies.

In ML, feature importance reveals the factors that are important to the model (42), whereas SHAP analysis clarifies the positive or negative contribution of the factors to the prediction equation (43). The results of feature importance analysis showed that sex, WC/Ht ratio, TG, and PG0 were important as predictive factors for SI, and the analysis of SHAP values showed that male sex displayed a positive impact, whereas WC/Ht ratio, BMI, and PG0 showed a negative impact on predicting SI in XGBoost. Previous studies have stated that fasting glucose and BMI have a strong influence as factors for SI estimation by ML (21–23), which is compatible with the results of the present study. A positive SHAP value indicates a contribution to increased insulin sensitivity, whereas a negative SHAP value indicates a contribution to decreased insulin sensitivity. Young men in the Jichi cohort displayed remarkably positive SHAP values (**Figure 2**), consistent with higher insulin sensitivity (**Table 1**). Higher BMI, WC/Ht ratio, and fasting glucose levels contributed to lower insulin sensitivity in the Hokuriku cohort (**Figures 3**, **4**), consistent with known pathophysiology.

Moderate correlations were observed between lipid-related estimates and SI, but these were not as strong as the correlations between ML-derived estimates and SI. Of the lipid-related estimates, the product of TyG index and physical indicators, as well as METS-IR, showed a relatively strong correlation with SI, consistent with earlier reports (16, 17, 19). Similarly, the correlation between lipid-related estimates and SI in the present study was not particularly robust. The results of the feature importance and SHAP analyses in the present study showed that the factors contributing to SI differed considerably depending on the background factors of age and glucose tolerance in the subject population. This suggests that it is difficult to create a universal and robust SI prediction equation simply by assigning fixed coefficients to conventional clinical parameters. Lipid-related estimates are calculated with fixed coefficients assigned to conventional clinical parameters. The main reason why lipid-related estimates do not universally correlate well with SI is that the contribution of various factors to SI differs according to subject background characteristics such as age and glucose tolerance.

A future follow-up study is needed to determine whether the SI estimates by ML in this study are useful in relation to future onset of metabolic syndrome and glucose intolerance in the young Jichi cohort, and future onset of cardiovascular events and cancer in the Hokuriku cohort.

## Limitations of the study

The limitations of this study are, first, that the SI prediction equations in ML are very complex. Although they showed good performance within each subgroup, they are hard to adapt to other subgroups as transfer learning. For example, when calculating the ISI-Matsuda for men in the Hokuriku cohort NGT using the XGBoost prediction equation (equations not shown) generated with seven factors for ISI-Matsuda in the Jichi cohort NGT men, the correlation coefficient (Spearman’s ρ value) with the actual ISI-Matsuda fell to 0.37. In practice, the clinical application of ML to SI prediction is complex because it requires analysis of each background factor. Second, this study did not obtain information on lifestyle habits such as exercise and diet that could contribute to SI. However, it has been reported that these factors are not significantly involved in prediction of SI by ML (21). Third, the presence of fatty liver is an important factor contributing to lower SI (29, 44, 45), but this could not be analyzed, because liver function test values were not available in the Jichi cohort. Fourth, this was a cross-sectional study and limited to Japanese participants. In this study, no external validation data beyond the Jichi cohort (young) and Hokuriku cohort (middle-aged) were included. In ML-derived SI estimates using conventional clinical parameters, it is necessary to take into account differences in race, age, sex, and glucose tolerance. Further external validation studies in diverse ethnic groups and also in subjects taking antidiabetic medications are needed. Fifth, in XGBoost in JMP Pro 17, the standard settings do not allow modification of resampling or random seeds. Therefore, it would be necessary to either change the statistical software or modify the JMP Pro 17 scripts to perform a reanalysis. Finally, feature importance and SHAP were adopted to interpret the XGBoost models in this study. Although XGBoost-derived estimates were robust within subgroups, their performance deteriorated when applied to another subgroup. There also remain possible biases on the results of feature importance and SHAP due to lack of lifestyle data, liver function test, and other unknown factors, such as menstrual status, contributing to SI.

## Conclusions

In Japanese young or middle-aged persons with NGT and middle-aged persons with GI, it was possible to estimate SI using ML based only on physical indicators, and by physical indicators together with lipid and fasting glucose levels. The contribution of each clinical factor to SI differed greatly by age and glucose tolerance status, implying that establishing robust estimates for SI by using conventional parameters would be difficult. Further validation studies are necessary in diverse ethnic groups with various body compositions.

## Data Availability

The datasets are available from the corresponding author on reasonable request. Requests to access these datasets should be directed to NM, norimitsu@med.showa-u.ac.jp.

## Supplementary material

Supplementary Figure 1 Calibration plots of ML indices by 3 factors for 1/HOMA-IR and ISI-Matsuda by sex in Jichi and Hokuriku cohorts. HOMA-IR, homeostasis model assessment of insulin resistance; ISI-Matsuda, Matsuda index; NGT, normal glucose tolerance; GI, glucose intolerance; MRA, multiple regression analysis; ANN, a neural network; DT, decision tree; RF, random forest; BT, boosting tree; KNN, K nearest neighbor; SVM, support vector machine; XGBoost, extreme gradient boosting.

Supplementary Figure 2 Calibration plots of ML indices by 7 factors for 1/HOMA-IR and ISI-Matsuda by sex in Jichi and Hokuriku cohorts. HOMA-IR, homeostasis model assessment of insulin resistance; ISI-Matsuda, Matsuda index; NGT, normal glucose tolerance; GI, glucose intolerance; MRA, multiple regression analysis; ANN, a neural network; DT, decision tree; RF, random forest; BT, boosting tree; KNN, K nearest neighbor; SVM, support vector machine; XGBoost, extreme gradient boosting.
