# Supplementary material for: Extreme gradient boosting using conventional parameters accurately predicts insulin sensitivity in young and middle-aged Japanese persons
Source: Front Endocrinol (Lausanne). 2025 Oct 17;16:1661376. doi: 10.3389/fendo.2025.1661376 (PMC12575152; doi:10.3389/fendo.2025.1661376)
Supplement: Supplementary file 3 [file Table1.docx]

Supplementary Table 1. Default hyperparameter settings of machine learning algorithms in JMP Pro 17

| Algorithm | Default settings | Validation method |
| --- | --- | --- |
| Multiple regression analysis (MRA) | No hyperparameter tuning; no internal validation by default | No validation by default |
| Artificial neural network (ANN) | Hidden layers: 1; Nodes per layer: 3; Learning rate: 0.1; L2 regularization applied; No early stopping | No validation by default |
| Decision tree (DT) | Iterative branching to maximize predictive performance; Pruned using cross-validation error and Akaike Information Criterion / Bayesian Information Criterion; Overfitting automatically reduced | Automatically selected depending on sample size |
| Random forest (RF) | Number of trees: 100; Variables per split: 2; Bootstrap sampling rate: 1; Splits per tree: max 2000, min 10; Minimum split size: 5 | Automatically selected depending on sample size |
| Boosted tree (BT) | Layers: ~5; Splits: 150–200; Learning rate: ~0.1; Minimum split size: 5; Parameters adjusted according to sample size and explanatory variables | Automatically selected depending on sample size |
| k-nearest neighbor (KNN) | K = 10; Category bias = 0.5 | Automatically selected depending on sample size |
| Support vector machine (SVM) | Cost parameter (C) = 1; Gamma = 1 / (number of explanatory variables); L2 regularization applied | Automatically selected depending on sample size |
| Extreme gradient boosting (XGBoost) | Maximum tree depth: 6; min_child_weight: 1; L1 regularization: none; L2 regularization coefficient: 1; Learning rate: 0.3; Number of boosting iterations: 30; No internal validation by default; Use all data and all features when building each tree | No validation by default |

Note: By default, none of the machine learning algorithms in JMP perform automatic early stopping. For algorithms with automatic validation, holdback validation (train/test split ratio: 70/30 split) was applied when the sample size was ≥500, and 5-fold cross-validation was applied when the sample size was <500.

Supplementary Table 2. R^2^, RMSE, and MAE of machine learning indices by 3 factors for 1/HOMA-IR and ISI-Matsuda by sex

|  | For 1/HOMA-IR | | | | | | For ISI-Matsuda | | | | | |  |  |  |
| --- | --- | --- | --- | --- | --- | --- | --- | --- | --- | --- | --- | --- | --- | --- | --- |
|  | Male | | | Female | | | Male | | | Female | | |  |  |  |
|  | R^2^ | RMSE | MAE | R^2^ | RMSE | MAE | R^2^ | RMSE | MAE | R^2^ | RMSE | MAE |  |  |  |
| Jichi cohort NGT (young) |  | | |  | | |  | | |  | | |  |  |  |
| MRA | 0.082 | 0.51 | 0.36 | 0.057 | 0.45 | 0.32 | 0.075 | 7.54 | 5.33 | 0.085 | 3.58 | 2.75 |  |  |  |
| ANN | 0.091 | 0.51 | 0.36 | 0.070 | 0.45 | 0.31 | 0.078 | 7.53 | 5.39 | 0.087 | 3.57 | 2.77 |  |  |  |
| DT | 0.43 | 0.40 | 0.27 | 0.37 | 0.35 | 0.25 | 0.48 | 5.50 | 3.81 | 0.42 | 3.21 | 2.33 |  |  |  |
| RF | 0.61 | 0.33 | 0.25 | 0.64 | 0.27 | 0.22 | 0.65 | 4.50 | 3.61 | 0.62 | 2.58 | 2.13 |  |  |  |
| BT | 0.64 | 0.32 | 0.24 | 0.36 | 0.35 | 0.27 | 0.66 | 4.43 | 3.46 | 0.46 | 3.10 | 2.39 |  |  |  |
| KNN | 0.14 | 0.49 | 0.35 | 0.15 | 0.41 | 0.30 | 0.18 | 6.94 | 5.01 | 0.14 | 3.89 | 2.87 |  |  |  |
| SVM | 0.12 | 0.51 | 0.34 | 0.075 | 0.44 | 0.28 | 0.12 | 7.35 | 4.96 | 0.13 | 3.48 | 2.44 |  |  |  |
| XGBoost | 0.85 | 0.20 | 0.18 | 0.97 | 0.078 | 0.074 | 0.82 | 3.24 | 2.74 | 0.97 | 0.67 | 0.63 |  |  |  |
| Hokuriku cohort NGT (middle-aged) |  | | |  | | |  | | |  | | |  |  | 0.18 |
| MRA | 0.063 | 1.89 | 0.81 | 0.037 | 1.57 | 0.63 | 0.010 | 15.09 | 7.93 | 0.13 | 8.14 | 5.28 |  |  |  |
| ANN | 0.059 | 1.89 | 0.81 | 0.025 | 1.58 | 0.32 | 0.11 | 15.02 | 7.74 | 0.11 | 8.24 | 5.45 |  |  |  |
| DT | 0.37 | 1.54 | 0.65 | 0.31 | 1.33 | 0.32 | 0.42 | 12.14 | 6.05 | 0.52 | 6.06 | 3.75 |  |  |  |
| RF | 0.51 | 1.37 | 0.62 | 0.47 | 1.17 | 0.23 | 0.54 | 10.83 | 5.72 | 0.60 | 5.53 | 3.75 |  |  |  |
| BT | 0.68 | 1.10 | 0.60 | 0.61 | 1.00 | 0.23 | 0.72 | 8.45 | 5.58 | 0.56 | 5.77 | 4.11 |  |  |  |
| KNN | 0.17 | 1.77 | 0.79 | 0.14 | 1.49 | 0.23 | 0.18 | 14.36 | 7.46 | 0.19 | 7.86 | 5.13 |  |  |  |
| SVM | 0.084 | 1.86 | 0.66 | 0.069 | 1.55 | 0.31 | 0.14 | 14.77 | 6.77 | 0.15 | 8.06 | 4.81 |  |  |  |
| XGBoost | 0.96 | 0.40 | 0.31 | 0.98 | 0.21 | 0.28 | 0.90 | 5.11 | 3.59 | 0.95 | 1.91 | 1.60 |  |  |  |
| Hokuriku cohort GI (middle-aged) |  | | |  | | |  | | |  | | |  |  | 0.021 |
| MRA | 0.033 | 1.61 | 0.65 | 0.31 | 0.43 | 0.32 | 0.13 | 5.88 | 3.84 | 0.088 | 5.67 | 3.11 |  |  |  |
| ANN | 0.030 | 1.61 | 0.67 | 0.32 | 0.42 | 0.32 | 0.12 | 5.90 | 3.88 | 0.086 | 5.68 | 3.13 |  |  |  |
| DT | 0.34 | 1.33 | 0.54 | 0.64 | 0.31 | 0.23 | 0.46 | 4.63 | 2.93 | 0.38 | 4.66 | 2.42 |  |  |  |
| RF | 0.54 | 1.11 | 0.51 | 0.68 | 0.29 | 0.23 | 0.56 | 4.16 | 2.82 | 0.56 | 3.92 | 2.32 |  |  |  |
| BT | 0.60 | 1.04 | 0.56 | 0.68 | 0.29 | 0.23 | 0.50 | 4.47 | 3.16 | 0.68 | 3.36 | 2.18 |  |  |  |
| KNN | 0.13 | 1.52 | 0.68 | 0.41 | 0.40 | 0.31 | 0.20 | 5.63 | 3.79 | 0.17 | 5.39 | 3.03 |  |  |  |
| SVM | 0.030 | 1.61 | 0.55 | 0.40 | 0.40 | 0.28 | 0.15 | 5.82 | 3.45 | 0.13 | 5.54 | 2.67 |  |  |  |
| XGBoost | 0.99 | 0.18 | 0.14 | 1.00 | 0.023 | 0.021 | 0.97 | 1.09 | 0.95 | 1.00 | 0.18 | 0.16 |  |  |  |

Abbreviations: R², Coefficient of Determination; RMSE, Root Mean Squared Error; MAE, Mean Absolute Error; HOMA-IR, homeostasis model assessment of insulin resistance; ISI-Matsuda, Matsuda index; NGT, normal glucose tolerance; GI, glucose intolerance; MRA, multiple regression analysis; ANN, a neural network; DT, decision tree; RF, random forest; BT, boosting tree; KNN, K nearest neighbor; SVM, support vector machine; XGBoost, extreme gradient boosting.

Supplementary Table 3. R^2^, RMSE, and MAE of machine learning indices by 7 factors for 1/HOMA-IR and ISI-Matsuda by sex

|  | For 1/HOMA-IR | | | | | | For ISI-Matsuda | | | | | |
| --- | --- | --- | --- | --- | --- | --- | --- | --- | --- | --- | --- | --- |
|  | Male | | | Female | | | Male | | | Female | | |
|  | R^2^ | RMSE | MAE | R^2^ | RMSE | MAE | R^2^ | RMSE | MAE | R^2^ | RMSE | MAE |
| Jichi cohort NGT (young) |  | | |  | | |  | | |  | | |
| MRA | 0.16 | 0.49 | 0.33 | 0.17 | 0.42 | 0.29 | 0.14 | 7.27 | 5.13 | 0.12 | 3.52 | 2.77 |
| ANN | 0.22 | 0.47 | 0.33 | 0.18 | 0.42 | 0.29 | 0.17 | 7.14 | 5.04 | 0.078 | 3.60 | 2.78 |
| DT | 0.52 | 0.37 | 0.25 | 0.50 | 0.31 | 0.20 | 0.55 | 5.12 | 3.37 | 0.54 | 2.84 | 2.01 |
| RF | 0.74 | 0.27 | 0.20 | 0.73 | 0.23 | 0.18 | 0.76 | 3.76 | 3.02 | 0.77 | 2.01 | 1.79 |
| BT | 0.76 | 0.26 | 0.20 | 0.49 | 0.31 | 0.24 | 0.79 | 3.52 | 2.75 | 0.51 | 2.94 | 2.33 |
| KNN | 0.27 | 0.45 | 0.31 | 0.20 | 0.40 | 0.29 | 0.21 | 6.80 | 4.79 | 0.18 | 3.80 | 2.75 |
| SVM | 0.31 | 0.44 | 0.28 | 0.33 | 0.38 | 0.22 | 0.26 | 6.73 | 4.24 | 0.34 | 3.04 | 2.10 |
| XGBoost | 0.91 | 0.16 | 0.13 | 0.99 | 0.038 | 0.032 | 0.91 | 2.27 | 1.85 | 0.99 | 0.41 | 0.36 |
| Hokuriku cohort NGT (middle-aged) |  | | |  | | |  | | |  | | |
| MRA | 0.089 | 1.86 | 0.78 | 0.054 | 1.56 | 0.61 | 0.15 | 14.72 | 7.72 | 0.19 | 7.87 | 5.02 |
| ANN | 0.083 | 1.87 | 0.76 | 0.043 | 1.57 | 0.59 | 0.16 | 14.60 | 7.36 | 0.17 | 7.95 | 5.12 |
| DT | 0.40 | 1.53 | 0.60 | 0.27 | 1.37 | 0.51 | 0.43 | 12.00 | 5.64 | 0.59 | 5.59 | 3.47 |
| RF | 0.58 | 1.26 | 0.55 | 0.47 | 1.16 | 0.46 | 0.61 | 9.90 | 4.97 | 0.73 | 4.55 | 3.02 |
| BT | 0.74 | 0.99 | 0.51 | 0.69 | 0.89 | 0.47 | 0.82 | 6.81 | 4.61 | 0.73 | 4.50 | 3.23 |
| KNN | 0.20 | 1.74 | 0.74 | 0.15 | 1.48 | 0.61 | 0.24 | 13.82 | 6.91 | 0.31 | 7.26 | 4.69 |
| SVM | 0.20 | 1.75 | 0.59 | 0.12 | 1.51 | 0.46 | 0.25 | 13.79 | 5.92 | 0.35 | 7.03 | 3.95 |
| XGBoost | 0.98 | 0.29 | 0.22 | 0.99 | 0.19 | 0.15 | 0.95 | 3.58 | 2.61 | 0.98 | 1.13 | 0.90 |
| Hokuriku cohort GI (middle-aged) |  | | |  | | |  | | |  | | |
| MRA | 0.068 | 1.58 | 0.65 | 0.46 | 0.38 | 0.28 | 0.15 | 5.81 | 3.79 | 0.11 | 5.60 | 2.99 |
| ANN | 0.036 | 1.61 | 0.66 | 0.46 | 0.38 | 0.29 | 0.11 | 5.97 | 3.94 | 0.13 | 5.52 | 3.08 |
| DT | 0.45 | 1.21 | 0.46 | 0.70 | 0.28 | 0.20 | 0.50 | 4.48 | 2.81 | 0.48 | 4.27 | 2.21 |
| RF | 0.57 | 1.08 | 0.46 | 0.80 | 0.23 | 0.19 | 0.66 | 3.70 | 2.49 | 0.63 | 3.59 | 2.02 |
| BT | 0.76 | 0.80 | 0.49 | 0.80 | 0.23 | 0.19 | 0.65 | 3.75 | 2.83 | 0.70 | 3.27 | 2.00 |
| KNN | 0.12 | 1.53 | 0.65 | 0.49 | 0.36 | 0.28 | 0.19 | 5.66 | 3.73 | 0.15 | 5.48 | 3.36 |
| SVM | 0.15 | 1.51 | 0.47 | 0.61 | 0.32 | 0.22 | 0.28 | 5.36 | 2.91 | 0.22 | 5.24 | 2.16 |
| XGBoost | 1.00 | 0.10 | 0.079 | 1.00 | 0.012 | 0.0086 | 0.99 | 0.60 | 0.52 | 1.00 | 0.033 | 0.027 |

Abbreviations: R², Coefficient of Determination; RMSE, Root Mean Squared Error; MAE, Mean Absolute Error; HOMA-IR, homeostasis model assessment of insulin resistance; ISI-Matsuda, Matsuda index; NGT, normal glucose tolerance; GI, glucose intolerance; MRA, multiple regression analysis; ANN, a neural network; DT, decision tree; RF, random forest; BT, boosting tree; KNN, K nearest neighbor; SVM, support vector machine; XGBoost, extreme gradient boosting.

Supplementary Table 4. Nonparametric Spearman rank correlation coefficients of lipid-related estimates for 1/HOMA-IR and ISI-Matsuda by sex

|  | For 1/HOMA-IR | | | | For ISI-Matsuda | | | |
| --- | --- | --- | --- | --- | --- | --- | --- | --- |
|  | Male | | Female | | Male | | Female | |
|  | ρ | *P* | ρ | *P* | ρ | *P* | ρ | *P* |
| Jichi cohort NGT  (young) |  |  |  |  |  |  |  |  |
| TG/HDL | -0.33 | <0.0001 | -0.19 | <0.01 | -0.33 | <0.0001 | -0.19 | <0.01 |
| LAP | -0.35 | <0.0001 | -0.22 | <0.001 | -0.31 | <0.0001 | -0.23 | <0.001 |
| VAI | -0.34 | <0.0001 | -0.20 | <0.001 | -0.34 | <0.0001 | -0.20 | <0.001 |
| DAI | -0.33 | <0.0001 | -0.20 | <0.001 | -0.34 | <0.0001 | -0.20 | <0.001 |
| TYG | -0.36 | <0.0001 | -0.22 | <0.001 | -0.32 | <0.0001 | -0.17 | <0.01 |
| TYG×BMI | -0.35 | <0.0001 | -0.24 | <0.0001 | -0.28 | <0.0001 | -0.19 | <0.01 |
| TYG×WC | -0.38 | <0.0001 | -0.25 | <0.0001 | -0.33 | <0.0001 | -0.23 | <0.0001 |
| TYG×WC/Ht | -0.38 | <0.0001 | -0.24 | <0.0001 | -0.36 | <0.0001 | -0.23 | <0.001 |
| AIP | -0.33 | <0.0001 | -0.19 | <0.01 | -0.33 | <0.0001 | -0.19 | <0.01 |
| METS-IR | -0.33 | <0.0001 | -0.26 | <0.0001 | -0.28 | <0.0001 | -0.22 | <0.001 |
| WTI | -0.34 | <0.0001 | -0.19 | <0.01 | -0.31 | <0.0001 | -0.19 | <0.01 |
| Hokuriku cohort NGT  (middle-aged) |  |  |  |  |  |  |  |  |
| TG/HDL | -0.40 | <0.0001 | -0.32 | <0.0001 | -0.40 | <0.0001 | -0.33 | <0.0001 |
| LAP | -0.53 | <0.0001 | -0.43 | <0.0001 | -0.50 | <0.0001 | -0.42 | <0.0001 |
| VAI | -0.41 | <0.0001 | -0.34 | <0.0001 | -0.41 | <0.0001 | -0.35 | <0.0001 |
| DAI | -0.40 | <0.0001 | -0.33 | <0.0001 | -0.40 | <0.0001 | -0.34 | <0.0001 |
| TYG | -0.39 | <0.0001 | -0.35 | <0.0001 | -0.39 | <0.0001 | -0.36 | <0.0001 |
| TYG×BMI | -0.56 | <0.0001 | -0.49 | <0.0001 | -0.52 | <0.0001 | -0.47 | <0.0001 |
| TYG×WC | -0.57 | <0.0001 | -0.46 | <0.0001 | -0.52 | <0.0001 | -0.45 | <0.0001 |
| TYG×WC/Ht | -0.55 | <0.0001 | -0.45 | <0.0001 | -0.53 | <0.0001 | -0.47 | <0.0001 |
| AIP | -0.40 | <0.0001 | -0.32 | <0.0001 | -0.40 | <0.0001 | -0.33 | <0.0001 |
| METS-IR | -0.55 | <0.0001 | -0.49 | <0.0001 | -0.51 | <0.0001 | -0.47 | <0.0001 |
| WTI | -0.43 | <0.0001 | -0.36 | <0.0001 | -0.42 | <0.0001 | -0.37 | <0.0001 |
| Hokuriku cohort GI  (middle-aged) |  |  |  |  |  |  |  |  |
| TG/HDL | -0.34 | <0.0001 | -0.48 | <0.0001 | -0.35 | <0.0001 | -0.45 | <0.0001 |
| LAP | -0.47 | <0.0001 | -0.55 | <0.0001 | -0.47 | <0.0001 | -0.48 | <0.0001 |
| VAI | -0.35 | <0.0001 | -0.49 | <0.0001 | -0.36 | <0.0001 | -0.46 | <0.0001 |
| DAI | -0.33 | <0.0001 | -0.48 | <0.0001 | -0.34 | <0.0001 | -0.45 | <0.0001 |
| TYG | -0.34 | <0.0001 | -0.49 | <0.0001 | -0.30 | <0.0001 | -0.46 | <0.0001 |
| TYG×BMI | -0.53 | <0.0001 | -0.61 | <0.0001 | -0.52 | <0.0001 | -0.47 | <0.0001 |
| TYG×WC | -0.51 | <0.0001 | -0.57 | <0.0001 | -0.50 | <0.0001 | -0.46 | <0.0001 |
| TYG×WC/Ht | -0.51 | <0.0001 | -0.55 | <0.0001 | -0.51 | <0.0001 | -0.45 | <0.0001 |
| AIP | -0.34 | <0.0001 | -0.48 | <0.0001 | -0.35 | <0.0001 | -0.45 | <0.0001 |
| METS-IR | -0.51 | <0.0001 | -0.62 | <0.0001 | -0.51 | <0.0001 | -0.48 | <0.0001 |
| WTI | -0.38 | <0.0001 | -0.51 | <0.0001 | -0.38 | <0.0001 | -0.47 | <0.0001 |

Abbreviations: HOMA-IR, homeostasis model assessment of insulin resistance; ISI-Matsuda, Matsuda index; NGT, normal glucose tolerance; GI, glucose intolerance; TG, triglycerides; HDL, high-density lipoprotein cholesterol; LAP, lipid accumulation product; VAI, visceral adiposity index; DAI, dysfunctional adiposity index; TYG, triglycerides and glucose index; BMI, body mass index; WC, waist circumference; Ht, height; AIP, atherogenic index of plasma; METS-IR, metabolic score for insulin resistance; WTI, waist-triglyceride index.
